# Salvarsannatrium

**Published:** 1916-02

**Authors:** 


**Salvarsannatrium** (No. 1206 A) has been developed at the suggestion of Ehrlich, Fabry and Fischer, Muench. Med. Woch., 1915, No. 18, page 612. This is freely soluble in water, free from hydraldite and is feebly alkaline in solution. It is dispensed in 0.4% salt solution in tubes containing 30, 45 and 60 centigrams, these being in single doses. The total volume of a single injection is 50-150 c. c. Intravenous administration is advised. The clinical phenomena resemble those of neosalvarsan, no severe symptoms having been noted in over 1,000 injections. The results, dosage, etc., also resemble those of neosalvarsan. This new preparation has a 20% arsenic content. Joint administration of mercury is advised. In this connection we have recently noted a suggestion by J. Alfred Cobb, Brit. Med. Jour., Dec. 11, to use a manometer cuff to bring out the veins of the elbow when they are small.

